# Collaborative clinical simulation in cardiologic emergency scenarios for medical students. An exploratory study on model applicability and assessment instruments

**DOI:** 10.3205/zma001472

**Published:** 2021-04-15

**Authors:** Sergio Guinez-Molinos, Carmen Gomar-Sancho

**Affiliations:** 1 Universidad de Talca, School of Medicine, Center of Clinical Simulation, Talca; Región del Maule, Chile; 2 University de Barcelona, Medical Faculty, Barcelona, Spain

**Keywords:** medical students' education, collaborative clinical simulation, tachyarrhythmia teaching, assessment instruments

## Abstract

**Aims: **This paper evaluates the feasibility of piloting the collaborative clinical simulation (CCS) model and its assessment instruments applicability for measuring interpersonal, collaborative, and clinical competencies in cardiologic emergency scenarios for medical students. The CCS model is a structured learning model for the acquisition and assessment of clinical competencies through small groups working collaboratively to design and perform in simulated environments supported by technology.

**Methods: **Fifty-five students were allocated in five sessions (one weekly session) conducted with the CCS model within the course Cardiovascular Diseases. The applied practice aimed at the diagnosis and treatment of tachyarrhythmias in a simulated emergency department. In addition to the theoretical classes four weeks before the simulation sessions, students were sent a study guide that summarized the Guide to the European Society of Cardiology. For each simulation session, one clinical simulation instructor, one cardiologist teacher, and the principal investigator participated. Students were divided into three groups (3-5 students) for each-session. They designed, performed, role-played, and debriefed three different diagnoses.

Three instruments to assess each group's performance were applied:

1. peer assessment used by groups,
2. performance assessment, created and applied by the cardiologist teacher, and
3. individual satisfaction questionnaire for students.

**Results: **The applicability of the CCS model was satisfactory for both students and teachers. The assessment instruments’ internal reliability was good, as was internal consistency with a Cronbach Alpha of 0.7, 0.4, and 0.8 for each section (Interpersonal, Clinical, and Collaborative competencies, respectively). The performance group’s evaluation was 0.8 for the two competencies assessed (Tachyarrhythmia and Electrical Cardioversion) and 0.8 for the satisfaction questionnaire's reliability.

**Conclusions: **The CCS model for teaching emergency tachyarrhythmias to medical students was applicable and well accepted. The internal reliability of the assessment instruments was considered satisfactory by measuring satisfaction and performance in the exploratory study.

## Zusammenfassung

**Ziele:** In dieser Arbeit wird die Durchführbarkeit der Pilotumsetzung des Modells für kollaborative klinische Simulation (KKS) und dessen Bewertungsinstrumenten für die Messung der interpersonellen, kollaborativen und klinischen Kompetenzen in kardiologischen Notfallszenarien für Medizinstudenten/-studentinnen ausgewertet. Das KKS-Modell ist ein strukturiertes Lernmodell für den Erwerb und die Bewertung klinischer Kompetenzen in kleinen Gruppen, in denen die Studenten/Studentinnen zusammenarbeiten und dabei die simulierten Umgebungen mithilfe von Technologie entwickeln und anwenden.

**Methoden: **Fünfundfünfzig Studenten/Studentinnen wurden fünf Sitzungen zugeordnet (eine Sitzung pro Woche), die unter Anwendung des KKS-Modells im Rahmen des Seminars Kardiovaskuläre Erkrankungen durchführt wurden. Dabei sollten die Diagnostik und Behandlung von Tachyarrhythmien in einer simulierten Notaufnahme geübt werden. Zusätzlich zum theoretischen Unterricht wurde den Studenten/Studentinnen vier Wochen vor Beginn der Simulationssitzungen ein Lernleitfaden mit einer Zusammenfassung der Leitlinien der European Society of Cardiology zugesendet. An den Simulationssitzungen nahmen jeweils ein Anleiter/eine Anleiterin im Bereich klinische Simulation, eine Lehrkraft für Kardiologie und der Studienleiter teil. Die Studenten/Studentinnen wurden für jede Sitzung in drei Gruppen (3–5 Studenten/Studentinnen) aufgeteilt. Sie entwarfen drei unterschiedliche Diagnosen, führten sie durch, wandten Rollenspiele an und führten ein Debriefing durch.

Zur Bewertung der Leistung jeder Gruppe wurden drei Instrumente angewendet:

1. Peer-Bewertung durch die Gruppen,
2. Leistungsbewertung, erstellt und angewendet durch die Lehrkraft für Kardiologie, und
3. Einzelfragebogen zur Zufriedenheit für die Studenten/Studentinnen.

**Ergebnisse: **Die Anwendbarkeit des KKS-Modells war sowohl für die Studenten/Studentinnen als auch die Lehrer/-innen zufriedenstellend. Die interne Reliabilität der Bewertungsinstrumente war gut, genau wie die interne Konsistenz mit einem Cronbachs Alpha von 0,7, 0,4 und 0,8 für jeden Bereich (jeweils Interpersonelle, Klinische und Kollaborative Kompetenzen). Der Wert für die Beurteilung der durchführenden Gruppe war 0,8 für die beiden bewerteten Kompetenzen (Tachyarrhythmie und Elektrische Kardioversion), und die Reliabilität des Zufriedenheitsfragebogens lag bei 0,8.

**Schlussfolgerungen: **Das KKS-Modell ist für die Lehre der Studenten/Studentinnen im Bereich Notfalldiagnostik und -behandlung von Tachyarrhythmien anwendbar und wurde gut angenommen. Die interne Reliabilität der Bewertungsinstrumente wurde durch Messung der Zufriedenheit und Leistung in der explorativen Studie als zufriedenstellend betrachtet.

## 1. Introduction

As future physicians, medical students will need to know how patient safety and teamwork impact the quality of healthcare [1]. These competencies are limited among medical curricula across various training levels, degrees, and specialties [2]. Students have few opportunities to learn how to be part of a medical team and learn from their own clinical mistakes [3].

Clinical simulation (CS) reduces the learning curves of the technical and attitudinal competencies that can be transferable to reality [3], [4]. Training non-technical competencies such as teamwork, leadership, situation awareness, and decision making [5] are essential to improving patient safety [6], [7], [8]. At the undergraduate student level, CS is increasingly used in medical schools, but it is usually oriented towards the acquisition of technical skills with phantoms or mannequins and "simulated" patient clinical interviews with actors [4], [9]. Nevertheless, suppose students are educated to become doctors through collaborative teamwork, the latter being framed by clinical practice [10]. In that case, clinical competencies should be achieved by simulating the environment of clinical procedures in real teams, learning through experience, and guided reflection [1], [11], [12].

As it’s used nowadays, CS presents several drawbacks for medical students that impede the expansion beyond the current uses [13]. However, these innovations are hardly replicable in other contexts because each teacher usually designs his or her simulation session from the beginning each time. Moreover, classical CS must be applied to small groups of 3-5 students [14]. However, when teachers must teach a large course, for example, one with 50-60 students, the teacher will have to repeat the same scenario ten or more times. This is not ideal because of the workload for the teacher and groups that repeat the scenario often already have the answers from their peers.

In a recent article [15], we have described a Collaborative Clinical Simulation (CCS) model to develop competencies for medical students. We defined it as the following: *“The structured learning phases for the acquisition, development, and assessment of clinical competencies through small groups learning collaboratively in the design, performance, and debriefing of simulated environments supported by technology”* [15].

The CCS model allows extending CS's advantages to 15 students in the same session, four to five times more students than recommended for classical CS. Furthermore, since all the students are exposed to three different clinical presentations, each featuring a distinct diagnosis, they get an almost 360º view of clinical competence by designing and solving the clinical scenario in small groups [15]. Within the process, the Collaborative Learning paradigm supports shared understanding and researches the interactions between participants in a learning activity [16]. We defended that the CCS model is applicable and affordable to medical schools that already use CS. The application of the CCS model is proposed as an adequate methodology to make diagnoses in acute situations and develop technical and non-technical skills in an acceptable timeframe with the clinical team. The medical students simulate the decision-making of clinical practice as a team with the context of a stressful and hyper-realistic emergency.

This article presents the feasibility pilot of the CCS model applied to the diagnosis and treatment of tachyarrhythmias in emergency cases. Moreover, we collected reliability data in the exploratory application for the assessment instruments associated with the CS scenario.

## 2. Material and methods

The CCS model's primary goals in the course “Cardiovascular diseases” were integrating theoretical learning and implementing a high-fidelity scenario. In this situation, students face an emergency scenario and within a team that have to coordinate, diagnose, and treat a patient suffering a tachyarrhythmia.

The medical students involved in this study were all doing their clinical rotation at the Hospital Clinic (Barcelona). Sample-size calculations were done by convenience, considering four-year medical students doing a clinical rotation and had the cardiovascular diseases’ theory classes completed.

The CCS’s applicability for the competence “Tachyarrhythmia Management in the Emergency Department” was structured following the CCS model described [15]. The educational objectives, materials, indices, case scenarios, and the simulation were designed, and the criteria for monitoring, rating, and debriefing were defined. The CCS model is structured in four stages (1. Educational design, 2. Students collaborative design, 3. Collaborative simulation, and 4. Debriefing). They are three types of participants (teacher, psychometrician, and students) and four different workspaces (academic unit, classroom, simulation room, and observation room) [15].

### 2.1. Stage 1 – educational design 

Firstly, the clinical goals were designed and coordinated between teachers from the medicine faculties at the Universidad de Talca (Chile) and Universität de Barcelona (Spain), and finished with scheduling the simulation sessions in the Clinical Skill Lab of the Faculty of Medicine at the Universität de Barcelona. The application was integrated into the course “Cardiovascular diseases”. The educational design and assessment instruments were created within the Universidad de Talca’s medical school and the Universität de Barcelona with experts on medical education and cardiology. Additionally, the Faculty of Psychology at the Universidad de Talca helped with the psychometrician analysis. The elaboration of the clinical guide for “electrical cardioversion” and “diagnosis and treatment of cardiac tachyarrhythmia” were delivered to students one month in advance for adequate study.

The session with the students started with a 30-minute introduction of the high-fidelity simulation. The students became familiar with the simulated “emergency room” environment, the simulated patient and all the available medical equipment and drugs as well as the available tests upon request. Confidence, personal safety, and respect among all participants were stressed in that phase.

#### 2.2. Stage 2 – students’ collaborative design of clinical scenarios 

**Figure 1:**
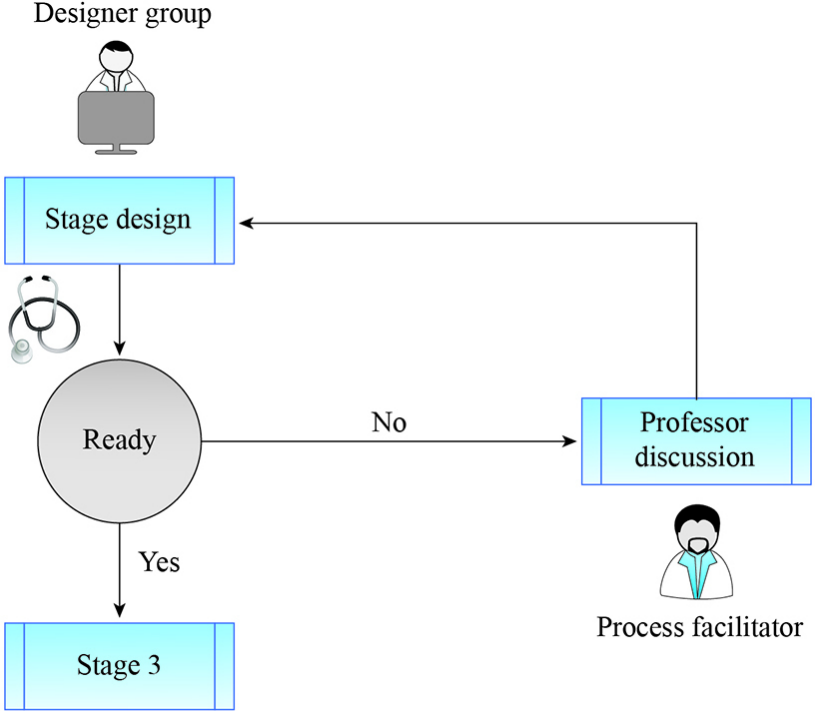
Collaborative design stage. Each group designs a clinical simulation scenario to apply to another group. The process is continuously discussed with a teacher (cardiologist), who reviews the case and gives feedback.

Three groups (3-5 students) were each allocated in different rooms. For each session, the participants were 12-15 students, the teacher, and one simulation instructor. Each group would begin by designing a clinical case centered on a differential diagnosis given by the instructor using EKG (e.g., Sinus Tachycardia, Atrial Fibrillation, Atrial Flutter, Paroxysmal Supraventricular Tachycardia, and Ventricular Fibrillation) which will be treated by another group, treating group. Working separately, each group will be given 60 minutes for the design of the simulated scenario, with roles, medical records, nursing sheets, and assessment, as well as free access to the Internet. To facilitate collaborative design, the instructor will provide standardized templates that include all the required information. The teacher will help each group design the clinical case (see figure 1 (Fig. 1)) to be consistent with a frequent clinical presentation of the tachyarrhythmia, including patient characteristics and even other factors such as relatives, the emotional status of the patient, etc. 

All the students’ background knowledge before the CCS sessions was from the theoretical classes in the course “Cardiovascular diseases” (malalties d’aparell cardiocirculatori, in Catalan), including electrocardiogram patterns, arrhythmia treatments, pharmacological treatment, and emergencies severity criteria. Moreover, these medical students had simulation’s experience in technical skills, but not with non-technical or teamwork (especially with a high-fidelity simulation scenario). 

#### 2.3. Stage 3 – collaborative simulation 

**Figure 2:**
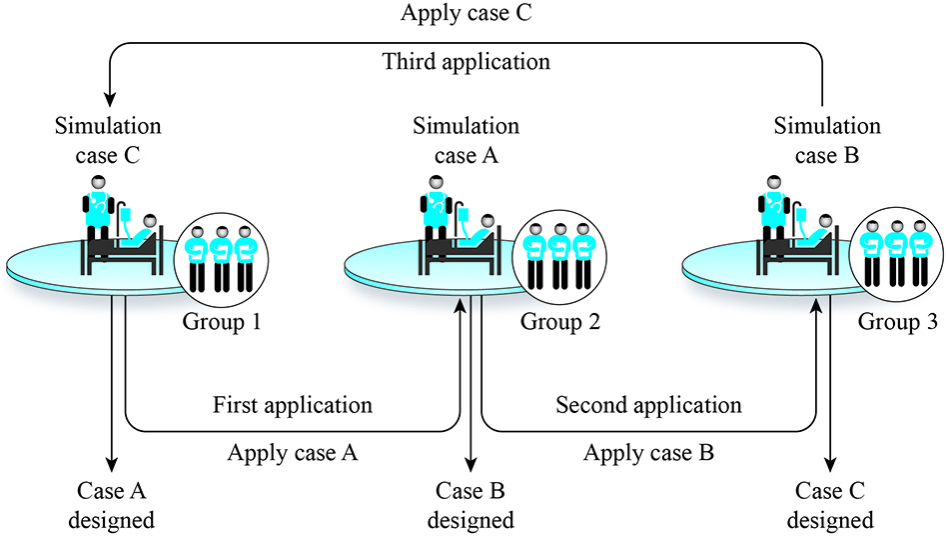
Collaborative Clinical Simulation. The clinical simulation represents a small group simulating the designed scenario to peers and assessing the simulation room’s performance. Case A: Atrial Fibrillation (AF), Case B: Paroxysmal supraventricular tachycardia (PSVT), Case C: Ventricular Fibrillation (VF).

The application of the designed scenarios was conducted in the simulation room of the Clinical Skills Lab with the simulator *SimMan* (Laerdal^®^), where each group applied the designed scenarios to peers (see figure 2 (Fig. 2)). This stage was composed of a sequence of three shifts. In the first shift, the designer group, (e.g., group 1) applied the simulation to another group, while the treating group (e.g., group 2) performed the first scenario. The designer group was responsible for controlling the simulator, its physiologic parameters, cameras, and workflow. Meanwhile, the observation group (e.g., group 3) observed the other two interacting groups from an observation room through a one-way mirror or video cameras. During this phase, the teacher was always assisting the designer group (in first shift) and assessing the treating group with the standardized rubric (created in stage 1, in collaboration between psychologists, a cardiologist, and medical education experts). In the second and third shifts, the roles of the groups changed (e.g., group 2 was designer, group 3 was treating, and group 1 was observing, and so forth). 

Before each group left the simulation room, the instructor remained alone with them, managing the group’s immediate emotions.

#### 2.4. Stage 4 – collaborative debriefing 

At the end of the three scenarios, all the participants met for structured reflection and discussion of the clinical scenarios applied. The instructors moderate the times and the focus of the debate. The debriefing stage was structured considering the sequence of the three shifts (stage 3). Each group received comments about their performance from the rest of the students and the instructor. Each case was discussed profoundly according to a structured plus/delta debriefing strategy [17], beginning by describing participant reactions followed by in-depth analysis, and ending with a discussion of the lessons learned [18]. Teamwork aspects and emotional management of the patient and his or her relatives were stressed. The teacher assured that the tachyarrhythmia emergency management was fully understood at the end of the session. 

**Figure 3:**
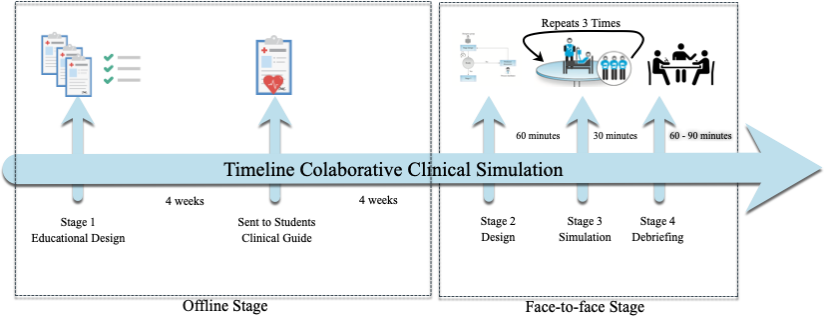
Collaborative Clinical Simulation Timeline: The timeline from Stage 1: Educational Design to Stage 4: Collaborative Debriefing.

The timeline for the CCS model’s were three months, considering the offline and online stages, showed in the figure 3 (Fig. 3). The total duration of stages 2, 3, and 4 (online of face-to-face stage) was approximately three hours.

#### 2.5. Assessment instruments

Psychometricians in collaboration with the teacher designed the assessment elements. In this exploratory study, reliability and validity data were collected to determine the quality of the assessment instruments.

Three instruments were created to assess the performance of each group and were applied (*in situ*):

1. peer assessment, used by groups (see table 1 (Tab. 1)),
2. satisfaction questionnaire for students (see table 2 (Tab. 2)), and
3. performance assessment (see table 3 (Tab. 3)), created and applied by the teacher.

The peer assessment applied by group members considers three areas:

1. interpersonal competencies: considers an appropriate personal treatment of patients/family,
2. clinical competencies: considers the correct clinical practice, and
3. collaborative competencies: considers the communication and collaboration inside the team.

Each one of these assessment instruments were carefully researched and structured to measure the non-technical skills involved in collaborative work. This work was conducted by simulation, psychometric, and clinical experts together and previously published [15].

The performance assessment instruments were designed and applied by a cardiologist, evaluating each group's performance in the clinical simulation scenario. The evaluation tool was created considering an emergency response performance tool as a reference [19]. The instruments applied were divided into two sections:

1. the managing of a tachyarrhythmia in the emergency room and
2. the electrical cardioversion procedure.

In the CCS method, when the designer group’s scenario is performed (collaborative simulation stage), they are responsible for assessing the treating group, according to the performance observed in the scenario. The satisfaction instrument was created to measure the students’ perceptions with the collaborative clinical learning environment. It is well known that students prefer to work in small groups, which promote positive participation and a perception of higher learning [20].

A five-point Likert scale was used to measure the peer assessment and the satisfaction instruments; scores ranged from 1 (minimum) to 5 (maximum) for all items.

Statistical methods can be used for different purposes [21]; the most common are validity and reliability analysis or estimate item difficulty separately for each item. These methods are specialized for determining the quality of any test, including the questions of an Objective Structured Clinical Examination (OSCE) [22], [23]. In this exploratory study, the Kaiser-Meyer-Olkin (KMO method [24], [25] was used to measure construct validity. Meanwhile the Cronbach Alpha [26] method was used to measure the reliability, and a descriptive analysis measured of the central tendency (min, max, mean) and measures of dispersion (variance, standard deviation).

All analyzes were performed with the software IBM SPSS Statistics 20

## 3. Results

Concerning the applicability of the model, the five sessions performed were run efficiently and timing adjusted. The time allowed students to advance and discuss the characteristics of a typical clinical presentation of tachyarrhythmia, apply the scenarios to other groups, and analyse the possible actions of the treating group and the responses of the patient to them. They self-assigned roles to play during the simulation scenario, such as a nurse, relatives, a senior doctor, and so on. The instructor was helping each group intermittently to program vital signs and responses for the simulator. 

### 3.1. Psychometrical analysis of assessment instruments 

The peer assessment instruments have three sections (A, B, and C), and psychometrics analysis considers each section separately. The instrument was filled out entirely by students who assessed the performance of their peers in the simulated scenario.

The KMO values in the the peer assessment instruments (see table 4 (Tab. 4)) exceeding the acceptable 0.6, and Bartlett's test of sphericity (Bartlett) [27] reached statistical significance (p<0.001). This showed a good correlation between the items and good sampling adequacy, respectively [28].

For interpersonal competencies, as indicated in the table 1 (Tab. 1) (see section A), the mean item scores ranged from a low of 3.37 for A5: “attended with appropriate speed to the clinical situation”, to 4.13 for A3: “responded to patient/family questions appropriately”. The scale’s reliability indicated by a Cronbach Alpha was 0.67 (see table 4 (Tab. 4)), and the item A3 was removed: “responded to patient/family questions appropriately”, moving up to 0.74.

The mean item scores in clinical competencies peer assessment ranged from a low of 2.80 for B2: “performed a correct physical examination”, to 4.80 for A4: “made the correct diagnosis (e.g., clinical reasoning)” (see table 1 (Tab. 1), section B). The scale's reliability indicated by a Cronbach Alpha was 0.36 (see table 4 (Tab. 4)). If item B5: “made the correct treatment (e.g., therapeutic plan, resolution)” were removed, it would be up to 0.50, showing unsatisfactory reliability results for this section.

The collaborative peer assessment (see table 1 (Tab. 1), section C) shows the mean item scores ranged from a low of 3.53 for C7: “the team shows a guide or leader among its members”, to 4.07 for C2: “the team shared and integrated knowledge”. The scale's reliability indicated by a Cronbach alpha was 0.57 (see table 4 (Tab. 4)). If are removed the items C3: “the team coordinated well your actions (e.g., integrated)” and C7: “the team shows a guide or leader among its members”, up to 0.82. Simultaneously, the KMO value would go up to 0.84, removing C3 and C7 items.

#### 3.1.1. The satisfaction questionnaire 

A satisfaction questionnaire was filled out for each student at the end of the session, including each stage of the CCS model applied (see table 2 (Tab. 2)). The questionnaire has two areas:

1. personal satisfaction with this activity and
2. usefulness of the different stages, with their perceived utility for their learning.

The mean of the items is high in all, approaching the maximum (5.0), while the variance is low (see table 3 (Tab. 3)), indicating that most students agreed with each of the reagents. That is, they were satisfied with the activity in general. The KMO value was 0.66, exceeding the acceptable 0.6, and Bartlett reached statistical significance (p<0.001), showing a good correlation between the items and good sampling adequacy. The discriminant function of reliability was good, as was internal consistency with the Cronbach Alpha of 0.77.

##### 3.1.2. The performance assessment

Table 3 (Tab. 3) (section A) shows the mean of the variables for managing a tachyarrhythmia in an emergency room. The highest mean of the dichotomous items corresponds to A1: “identifies the heart rate” with 0.87, and the lowest was item A6: “identifies hemodynamic instability” with 0.60. The scale's reliability indicated by Cronbach Alpha was 0.78, considered acceptable, and if the dichotomous item A7: “indicate the need for electrical cardioversion” were removed, it would be up to 0.81.

In table 3 (Tab. 3), section B, the electrical cardioversion procedure, 13 dichotomous items have a total reliability of .79. The lowest mean, and therefore the most deficient performance of the students, was in item B4: “verifies permeable venous line” with 0.13. On the contrary, the groups’ best performance was obtained in items B7: “preoxygenation with Ambu-Oxygen 100%” and B9: “charge defibrillator in synchronous mode” with a mean of 0.87 and 0.80, respectively. 

## 4. Discussion and future work

Multiple authors agree that CS supports the acquisition and development of clinical competencies for medical students inside realistic scenarios [29], [30], [31]. However, at the undergraduate students level, CS is used frequently to develop individual competencies [30], [32], which will not be the reality of a professional clinical environment. In this context, it is essential to teach medical competencies to students within a team and in collaborative environments [10], [32], incorporating these innovative methodologies formally into the curriculum [6], [17], [33], stimulating teamwork and patient safety [34]. Conventional CS is applied to small groups, 3-5 participants in each session. That presents a significant limitation for use with large groups of students in medical schools. Those facts greatly limit the extension of CS in medical schools and impede teaching clinical competencies as they develop in the real medical practice. 

The CCS exploratory application results were satisfactory; the applicability of the method is correct, and that there is internal reliability of your assessment methods. Within a formal exploratory analysis, the reliability value around 0.7 is adequate and is the minimum acceptable level [35]. In the early phases of research or exploratory studies, a reliability value of 0.6 or 0.5 may be sufficient [36]. Loewenthal [37] suggests that a reliability value of 0.7 can be considered acceptable for scales with less than ten items. 

The satisfaction perceived by students was high. They unanimously proposed to continue and formally extend the model CCS to several clinical materials of the curriculum. The medical students rated positively, with a mean of 4.98, “The attention given by the teachers in the simulation” and “The reflect on the clinical case in the debriefing” moderated by teachers.

The use of numerous evaluation tools in an emergency scenario was not a problem for the model's applicability. They were well distributed and in different stages. In the scenarios’ execution, the groups applied the peer assessment instruments; in parallel, the cardiologists measured the treating group's performance. At the end of all the scenarios, the participants filled out the satisfaction questionnaire in the debriefing stage. The whole process was fluid, and there were no significant complications.

The applicability of the CCS model was satisfactory for teachers, too. With its structured order (3 hours per session, 15 students, 3 cases with all students participating in some way), it was considered more efficient than the classical CS and without apparent difficulties to be extended in other medical schools. The CCS was used for teaching tachyarrhythmias management in three clinical presentations, including diagnosis, emergency treatment with drugs and cardioversion, and attention to the patient and relatives’ emotional status. This was all done within a team in 3 hours for 15 students and was very efficient compared to the classical CS. Technical and non-technical competencies are acquired simultaneously in the CCS model. Furthermore, the competencies required for students to participate in clinical scenarios is more practical than in an academic-only environment and would allow clinicians to participate more actively in teaching the curriculum.

The medical students who participated in the study had completed their theoretical classes within the course “cardiovascular diseases”. However, the scarce clinical practice was evidenced in lower scores for item 7: “the team performed a correct physical examination” (clinical skills, section B). Moreover, managing tachyarrhythmia in a simulated emergency room was successful. However, the electrical cardioversion procedure had several lower score items (bed in a horizontal position, verifies permeable venous line, verifies whether the patient is spontaneously ventilating, and applies oxygen mask) that did not exceed 0.3 on average.

It was the first time the group of participants faced a simulated emergency with cardiologic emergency scenarios. They had to make decisions and apply knowledge, going from being passive spectators to protagonists of the situation. This was undoubtedly valued well, and the degree of satisfaction with the model was high.

No significant differences were observed between groups since all of them came to the study with their theoretical classes and studied the guides provided by the teachers specially designed for this practice. Besides, students in general positively evaluated adding teamwork skills to clinical skills. This shows the applicability of the CCS model to develop non-technical skills.

The CCS’s limitations are centered on demonstrating evidence that the model effectively develops the competencies (clinical and non-technical skills of tachyarrhythmias management in the emergency department). In the exploratory application of CCS described in this paper, we have demonstrated both easy applicability, efficiency (time and specialists’ hours), and internal reliability to the primary assessment instrument. Evidence of efficacy of any teaching method in medicine, including CS, is always complicated since it would require observing the individual applying that competence in the clinical practice. Research on reliable tools to assess the efficacy of teaching must be developed. Furthermore, although CCS performs better than CS to acquire both clinical and non-technical skills and allows them to teach more profoundly and to more students, we require additional studies for validity. We are considering using one station of the Objective Structured Clinical Examination (OSCE) [23], [38] dedicated to tachyarrhythmias management for differentiating the competence in students receiving CS or CCS methodology. 

Medical schools should consider integrating teamwork skills when teaching clinical competencies and making an analysis (or prospective evaluation) to judge how much teaching about teamwork currently exists and how much is needed [1]. 

Considering this, it is essential to reflect on the advantages of CCS for teamwork [39] and integrating collaborative learning [40] into CS. Its application for the diagnosis and treatment of tachyarrhythmias in a simulated emergency department was satisfactory for students and teachers, measuring assessment instruments with reliability and validity based on statistical analyses [29], [41], [42], [43].

## Competing interests

The authors declare that they have no competing interests. 

## Figures and Tables

**Table 1:**
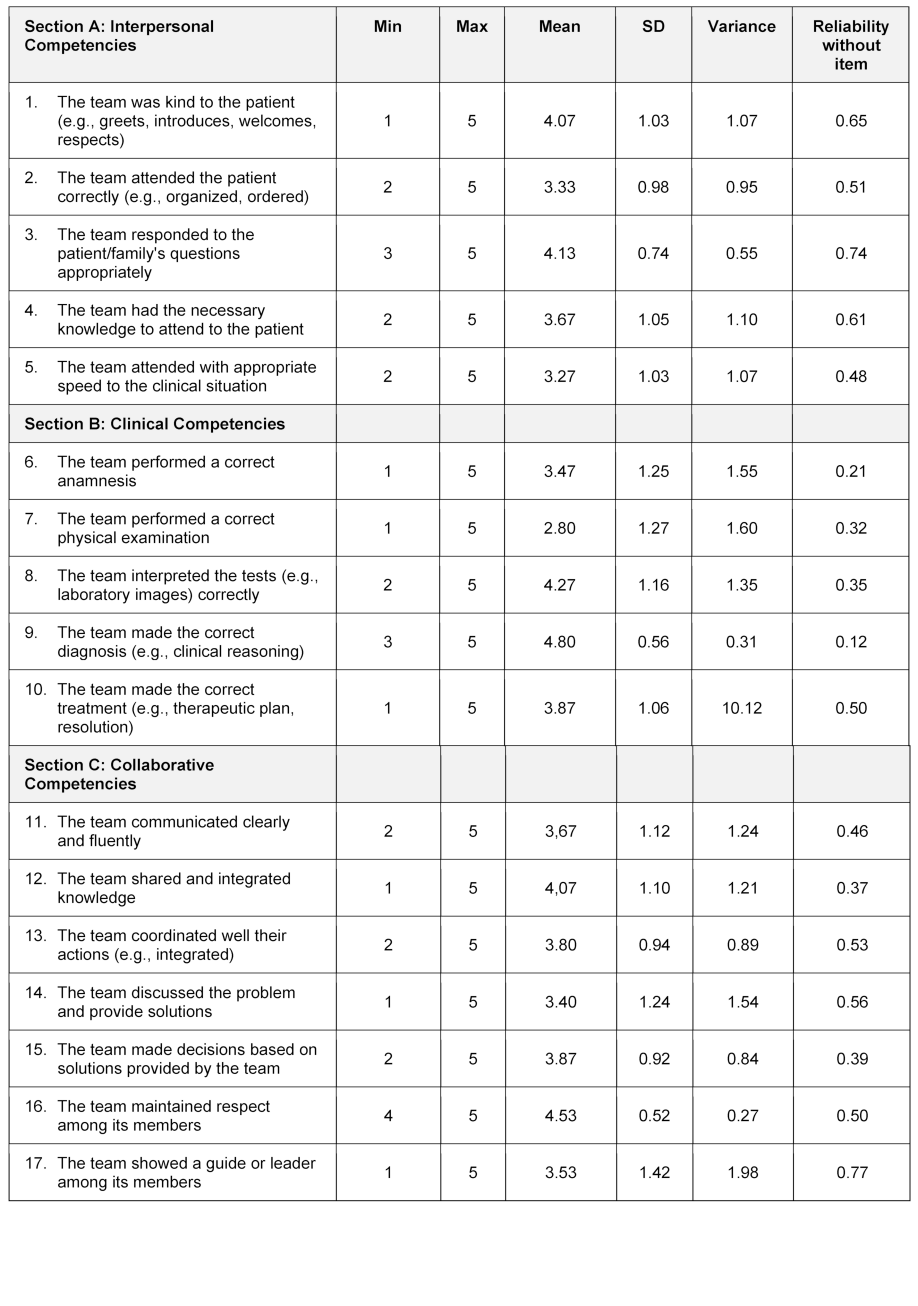
Peer assessment instrument: Instruments applied by "instructor group," who assess the "treating group" performance in the simulated scenario. A five-point Likert scale was used to measure; scores ranged from 1 (minimum) to 5 (maximum) for all items.

**Table 2:**
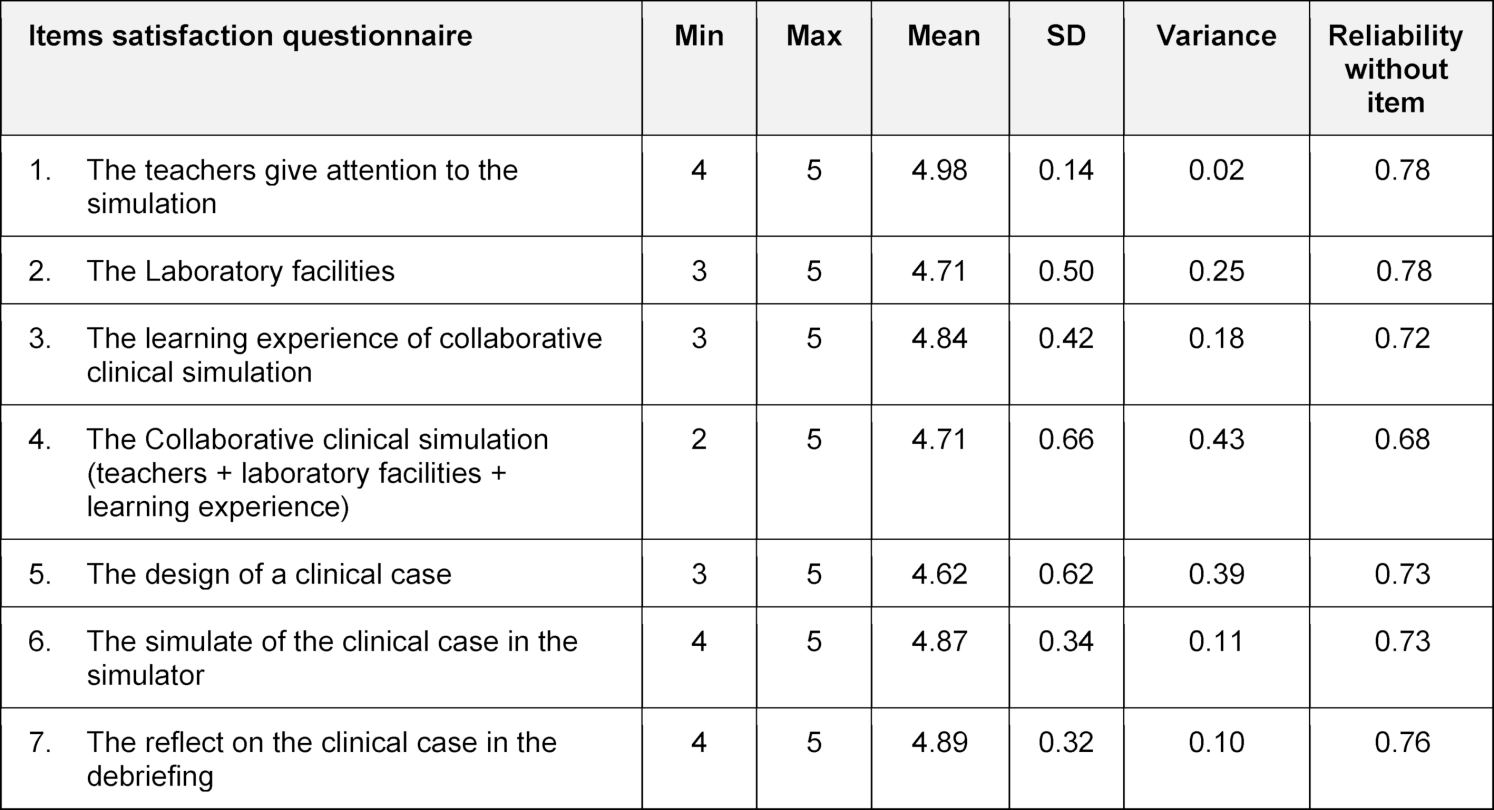
The satisfaction questionnaire: The instruments have two dimensions to measure satisfaction. The general aspects (teachers, laboratory, and learning experience) and each of the CCS model stages. A five-point Likert scale was used to measure; scores did range from 1 to 5.

**Table 3:**
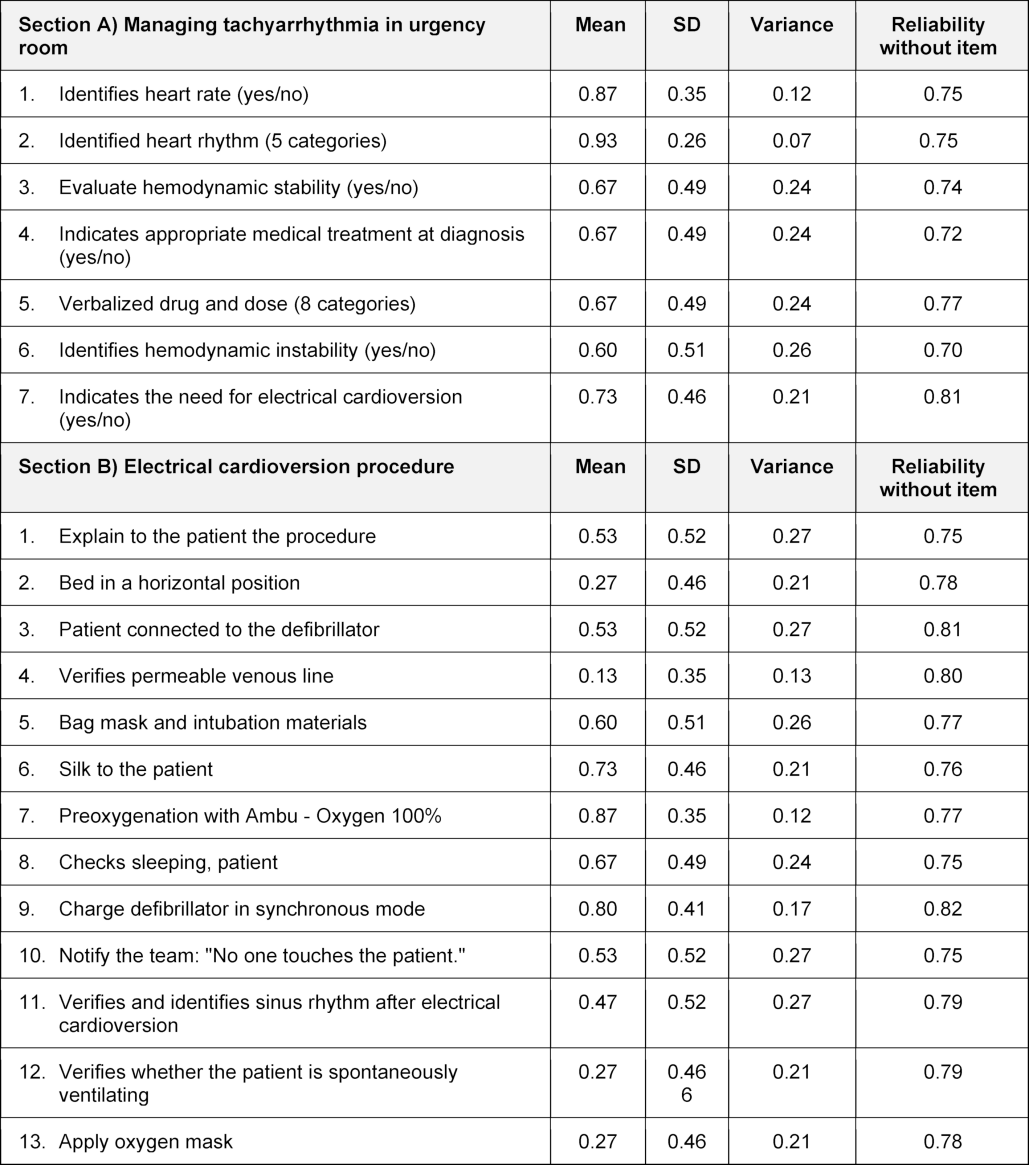
Group Performance Instruments. Divided into two sections: A) Managing tachyarrhythmia in urgency room and B) Electrical cardioversion procedure, with dichotomous items to assess the group's performance in the simulated scenario.

**Table 4:**
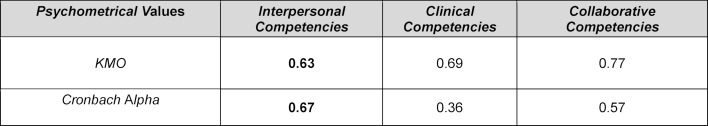
Psychometrical value for peer assessment instruments. Kaiser–Meyer–Olkin (KMO) [24], [25] was used to measure construct validity, and Cronbach Alpha [26] to measure the reliability.
